# The D2Refine Platform for the Standardization of Clinical Research Study Data Dictionaries: Usability Study

**DOI:** 10.2196/10205

**Published:** 2018-07-25

**Authors:** Deepak Kumar Sharma, Kevin Jerrold Peterson, Na Hong, Guoqian Jiang

**Affiliations:** ^1^ Department of Health Sciences Research Mayo Clinic Rochester, MN United States

**Keywords:** usability study, data dictionary, interoperability, electronic health records, usability framework, metadata, standardization

## Abstract

**Background:**

D2Refine provides a Web-based environment to create clinical research study data dictionaries and enables standardization and harmonization of its variable definitions with controlled terminology resources.

**Objective:**

To assess the usability of the functions D2Refine offers, a usability study was designed and executed.

**Methods:**

We employed the TURF (task, user, representation, and function) Usability Framework of electronic health record usability to design, configure, and execute the usability study and performed quantitative analyses. D2Refine was compared for its usability metrics against two other comparable solutions, OntoMaton and RightField, which have very similar functionalities for creating, managing, and standardizing data dictionaries. We first conducted the function analysis by conducting one-on-one interviews armed with questionnaires to catalog expected functionality. The enrolled participants carried out the steps for selected tasks to accomplish specific goals and their feedback was captured to conduct the task analysis.

**Results:**

We enrolled a group (n=27) of study developers, managers, and software professionals to execute steps of analysis as specified by the TURF framework. For the within-model domain function saturation, D2Refine had 96% saturation, which was 4 percentage points better than OntoMaton and 28 percentage points better than RightField. The manual examination and statistical analysis of the data were conducted for task analysis, and the results demonstrated a significant difference for favorability toward D2Refine (*P*<.001) with a 95% CI. Overall, 17 out of 27 (63%) participants indicated that D2Refine was their favorite of the three options.

**Conclusions:**

D2Refine is a useful and promising platform that can help address the emerging needs related to clinical research study data dictionary standardization and harmonization.

## Introduction

The process of creating and managing interoperable metadata is challenging. Additionally, the use of spreadsheets or simple tabular forms to express and organize metadata definitions is widespread in the research community. The environments with spreadsheets and tabular interfaces are common, simple, and flexible, and familiarity with creating content in them reduces the learning curve considerably. The trend, toward the ability to manage metadata using the simple tabular interface of a spreadsheet, is also evident from the list of metadata tools identified by Stanford University Libraries [1]. These solutions have nontrivial installation, configuration, and workflow steps to create and manage metadata. The translation of metadata is usually proprietary and does not adhere to a standard format, reducing interoperability. A standard representation of metadata would assist metadata developers to identify a minimal core set of information and help create metadata models with enhanced interoperability and shared semantics. In our ongoing studies [2], D2Refine Workbench [3] (D2Refine for short) is being developed to address these issues and to make the process of creating metadata easier using a simpler interface and disseminated models with enhanced interoperability. This greatly reduces the complexity, learning curve, and additional documentation and transformation steps that would otherwise be needed to make models usable outside their local context, when shared.

D2Refine is built on top of an open-source solution called OpenRefine [4] (formerly known as Google Refine), which offers a simpler, spreadsheet-like interface. D2Refine leverages the extensible OpenRefine framework to augment customizable services to create terminology bindings for standardization efforts. It extends export mechanism to serialize models into standard formats to persist and share with other metadata developers. Our objective was to design and conduct a usability study, following the proven methodology of the TURF (task, user, representation, and function) framework of electronic health record (EHR) usability [5], to assess the usability and usefulness of D2Refine platform. The TURF framework comprises four analysis areas of focus to facilitate designing and conducting an effective usability study. The TURF framework helps us gauge three aspects of a system: useful (ability to support the work domain), usable (easy to learn, use, and error-tolerant), and user satisfaction (likability). The TURF framework guidelines for function and task analyses were employed in this usability study for D2Refine. The novelty of this usability study was that it selectively adopted the TURF framework’s systematic and nonconfounding function and task analyses guidelines for identifying comparable features of an environment against one or more competing environments. The applicability of the TURF framework guidelines saved us time and effort related to setting up our own guidelines and processes; we instead focused on evaluating environments. Two comparable open-source solutions, OntoMaton [6], developed by Investigation, Study, and Assay (ISA) Tools using the ISA framework, and RightField [7], developed by University of Manchester, were selected for side-by-side comparison with D2Refine. The choices of comparable environments came from our own knowledge about these systems and from a list of metadata tools created by Stanford University Libraries [1]. OntoMaton is a set of plugins to Google Sheets that allows users to manage and standardize data dictionaries, whereas RightField offers similar capabilities through a Java library programmed to work with Microsoft Excel. RightField uses Apache POI Library [8] and Protégé Web Ontology Language (OWL) API [9] to work with spreadsheets and ontologies, respectively. Since all three environments present very similar interfaces (spreadsheet or spreadsheet-like) to clinical study developers, the user analysis and representation analysis aspects of the TURF framework were deferred and not included in this usability study.

This paper describes the requirements collection, execution, and results of the usability study. It includes the selection and results of metrics for function analysis and quantitative evaluation through task analysis. While the function analysis provides insights into the usefulness of D2Refine, the task analysis throws light on the usability of D2Refine for a selected set of tasks. The task analysis is extended to quantify the satisfaction level of the participants, who completed the selected set of tasks.

## Methods

### Study Design

D2Refine, OntoMaton, and RightField offer viewing, standardizing, and serializing capabilities for data dictionaries with their simple tabular interfaces. In the following subsections, we briefly introduce them and describe their comparable capabilities that are included in the usability study. The TURF framework is the guiding element in this usability study and as we introduce the relevant aspects of the framework. We also describe the participants, who are the most important part of this study.

### Materials

#### D2Refine Workbench

**Figure 1:**
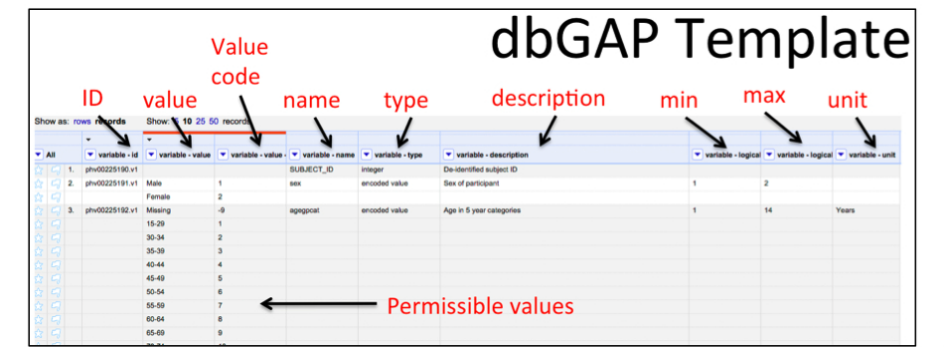
A dbGaP Data Dictionary viewed in D2Refine. The column headers describe the metadata elements (variable id, name, type, etc) of a data dictionary. dbGaP: database of Genotypes and Phenotypes.

As mentioned above, D2Refine is developed by extending an open-source platform, OpenRefine, to help clean up and organize data in an intuitive manner to anyone who is conversant working with spreadsheets. This greatly reduces the learning curve as D2Refine allows a user to create data dictionaries simply by arranging data dictionary variable definitions as rows. In addition, D2Refine leverages the OpenRefine’s capability to directly import and ingest content directly from Web-based data dictionaries such as those from the database of Genotypes and Phenotypes (dbGaP) [10]. Figure 1 shows a data dictionary in D2Refine that is imported directly from dbGaP (by using the web address of the data dictionary). The dbGaP metadata elements are marked to demonstrate how simply D2Refine processes and represents them to the user.

D2Refine further extends the built-in reconciliation service mechanism of OpenRefine to standardize the data dictionary variables. D2Refine can add and utilize any Common Terminology Services 2 (CTS2) [11]-compliant terminology service to search and link terms to the data dictionary variable definitions. The D2Refine workbench comes preconfigured with a default reconciliation service, the National Cancer Institute’s (NCI) Lexical Enterprise Vocabulary System (LexEVS) CTS2 Service [12], which provides a quick-start for users to standardize the data dictionary content. D2Refine’s export or import extensions provide a way to serialize content to a desirable standard or customized format. Although D2Refine implements an extension to serialize a data dictionary to openEHR’s Archetype Definition Language (ADL) [13] format, its evaluation for usability was not included in this study.

#### OntoMaton

The OntoMaton Google Widget, developed by ISA-Tools [14], is a plug-in widget that works with Google Spreadsheet [15] documents. Once this widget is augmented to Google Spreadsheet as an add-on, it can be invoked to display a right-hand side panel that shares screen with the spreadsheet. OntoMaton lets users connect and search biomedical terms from the National Center for Biomedical Ontology’s Bioportal [16,17], Linked Open Vocabularies [18], and Ontology Lookup Service [19].

**Figure 2:**
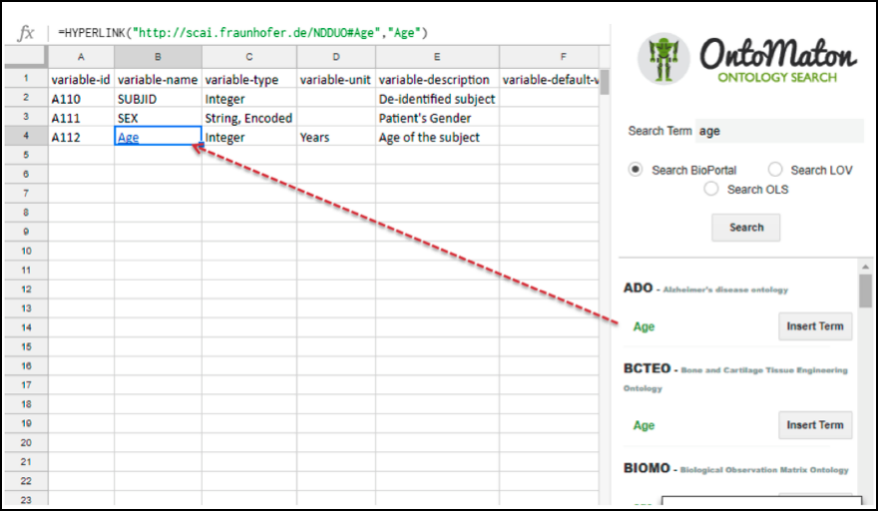
The OntoMaton Ontology Annotator works with Google Spreadsheets as an add-on to a user’s Google Spreadsheets account.

OntoMaton allows searching with key-phrases in individual and batch mode and organizes by grouping the result candidates, in an effort to help users select an appropriate match and create a terminology (term) binding (linking a cell value to a reference of a controlled vocabulary term; Figure 2). These term bindings are stored as additional worksheets as part of the user’s original data dictionary definition spreadsheet.

#### RightField

**Figure 3:**
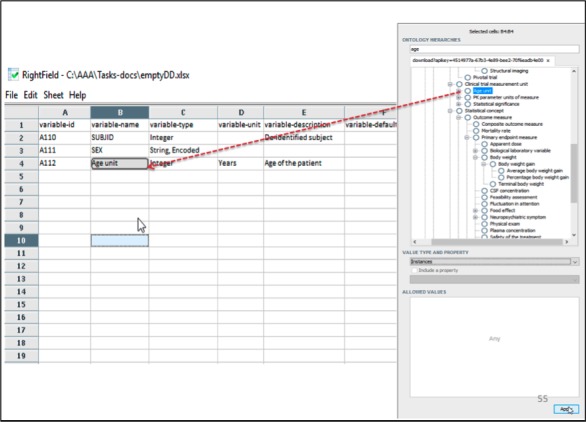
A term binding in the RightField Ontology Term Annotator.

RightField is another open-source dataset annotation tool developed by the University of Manchester [20] and the Hiedelberg Institute for Theoretical Studies [21] (Figure 3). It is designed to work as a standalone Java application, which uses Apache’s POI [8] to manage data dictionary content in and as Microsoft Excel documents. Similar to OntoMaton, RightField opens up a right-hand side panel with a selected set of ontologies to search and select. A user can create or open an existing Microsoft Excel document to work with RightField and standardize the content. RightField is programmed to load multiple ontologies to work with, although it slows down the lookup and sometimes adversely alters the ontology hierarchy tree rendering.

The RightField Ontology Term Annotator allows user to create term bindings for a cell value with a selected matched term from the ontology (illustrated in Figure 3 with a dashed arrow). RightField lets users store a term binding along with a constraint to validate instance data, by allowing the choice of either a class hierarchy or an instance of the matched ontology term. RightField, similar to OntoMaton, manages the term bindings by augmenting auxiliary worksheets to the user’s spreadsheet.

#### The TURF Framework of EHR Usability

The TURF Framework of EHR Usability guided the implementation of a usability study to compare environments and defined the ways of measuring the dimensions of usability for each. We used the TURF framework to assess the usefulness of a system by employing the function saturation metrics to help portray coverage of the useful functional capabilities. The task analysis helped us measure efficiency and robustness in task completion workflows and helped us understand the efforts required to accomplish user goals, error prevention, and recovery and learnability, that is, how usable the system was for doing the work. Additionally, the satisfaction level of users could be captured with surveys, interviews, and questionnaires attached to the various tasks users completed over the course of the study.

### Methods of Usability Study

Following institutional review board approval and participants’ provision of informed consent, we enrolled 27 participants. Out of these, 15 participants were clinical study developers and 12 participants were a mix of administration and information technology professionals, who develop and support applications for health sciences research at Mayo Clinic, USA. Most of the study developers who participated already used various applications to create, manage, and disseminate clinical study artifacts. For example, some of them were responsible for creating case report forms [22], which are equivalent to data dictionary definitions. These case report forms are composed together, similar to data dictionaries, to design and conduct studies for various domains of healthcare research. Many of these workflows included referencing- and linking-controlled terminologies for their list of terms and codes. Enrolled participants were invited and vetted for their knowledge and familiarity with the clinical study data dictionaries. Employing the TURF framework for evaluating the usability of D2Refine hinged on recording and learning from the experiences of the participants as D2Refine was compared with OntoMaton and RightField. This study was a combination of two types of analyses: the function analysis and the task analysis. The data gathered helped us quantitatively identify the usability strengths and weaknesses of one system over another, which made it easier to state our conclusions for each system involved.

#### Function Analysis

The idea of the function analysis is based on measuring the usefulness of a system by its implementation of essential functions. Function analysis helps identify the implementation of critical functions, without which a system will fail. One of the initial steps of function analysis is to identify the functions and the work domain under which they fall. The functions fall in at least one of the three categories: (1) Functions that a system implements, (2) Functions that users want, and (3) Functions that actually get used to carry out the tasks to accomplish goals. The TURF framework describes these categories as three models: the Designer Model, User Model, and Activity Model, respectively. The level of usefulness of a system is proportional to the overlapped regions of these three models. The TURF framework recommends organizing the identified functions into a work domain ontology. The collected functions are further evaluated to filter nonessential functions from critical functions.

##### The Questionnaire Design for the Function Analysis

Awareness about the functions that fell into the three models of the TURF framework yielded creation of an effective and useful work domain ontology. This work domain ontology clearly depicted the functional coverage of participating environments. To catalog the desired functionality (and weigh them against implemented and used functions), we designed a questionnaire for the study participants. This questionnaire queried participant’s existing environments as well as what functionalities they wanted to see implemented in a solution. They were quizzed about ways in which they created, stored, and disseminated dataset definitions and their use of controlled vocabularies. The questionnaire included multiple-choice questions as well as questions for detailed free text responses. A careful capture of information from participants proved useful in listing their problems, expectations, and recommendations for an ideal environment.

##### The TURF Metrics

**Figure 4:**
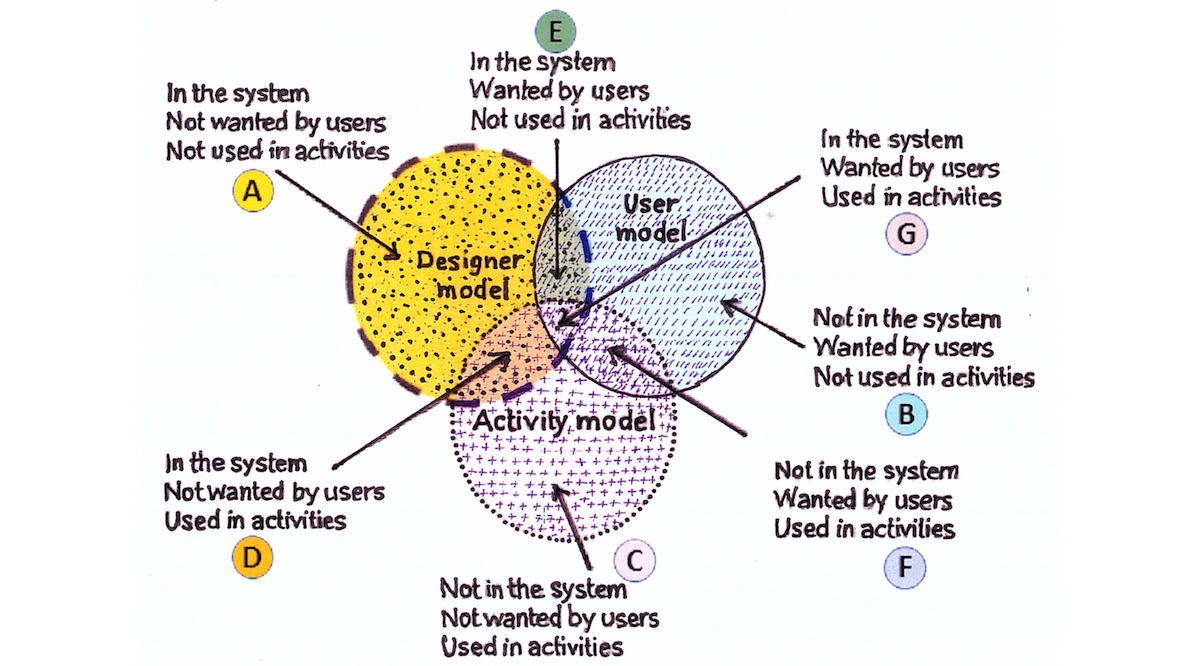
The Venn diagrams of the TURF (task, user, representation, and function) Framework’s domain models are labeled to help understand the domain function saturation metrics computation.

We computed two function saturation metrics of the TURF framework to assess usefulness. The Venn diagrams of TURF framework’s Domain Models [23] in Figure 4 are ways of understanding the coverage (the overlapped areas among the three models) of the functions. The metrics describe the quantitative overlapped portions of implemented functions in an environment (Designer Model) that the user wanted (User Model), and which were eventually used during activities (Activity Model) to accomplish a task.

Within-model domain function saturation: This metric quantifies the functional coverage by the current implementation of functions and is computed as the ratio of domain functions to total functions in the Designer Model. This is calculated as the sum of the number of functions in the regions of D, E, and G divided by the sum of the number of functions in the regions of A, D, E, and G (Figure 4). The number of functions in regions A will be inversely proportional to the domain function saturation or coverage.

Across-model domain function saturation: This is the ratio of domain functions in the Designer Model to the domain functions in all three models (Designer, User, and Activity). It is calculated as the sum of the numerators of the fractions in the regions of A, D, E, and G divided by the count of all domain functions in all three models (Figure 4).

The user questionnaire, discussed in the previous section, was instrumental in capturing the domain functions in these three models. An OWL [24] work domain ontology was created to persist and gain statistical insight into the list of uniquely identified functions. This ontology had a number of top-level OWL classes to partition functions into three models: Design, User, and Activity. Each identified function descended from these top-level classes and each instance of such class related to its implementation in one or more environments. Arranging the classes and instances this way allowed us to quickly and programmatically compute TURF metrics for functional coverage. A set of scripts in Python [25] were developed to query the work domain ontology by using SPARQL Protocol and RDF Query Language [26]. These utility scripts were also used to dynamically create Venn diagrams to illustrate results for better understanding.

#### Task Analysis

The TURF framework describes task analysis as the process of identifying the steps (mental and physical) and their interdependence to carry out an operation by using a specific representation in an environment. In this usability study, the task analysis steps that the participants followed were relatively simple and straightforward. Since all three environments used very similar spreadsheet-like interfaces, the task analysis focused on fair comparison of performing few selected tasks in each of the environments. The order of selecting the environments to perform tasks was random to avoid bias. Each participant was provided the introduction and appropriate documentation to the three testing environments, D2Refine, OntoMaton, and RightField, in a separate tutorial session. The tutorial sessions were conducted to make participants familiar, comfortable, and conversant in the performance of tasks to accomplish goals of creating, editing, persisting, and standardizing data dictionary elements.

In addition to providing verbal feedback, the participants answered two questions for each task performed in each of the environments. These two questions captured their level of satisfaction using a 5-Point Likert scale [27] (strongly disagree, disagree, neither agree nor disagree, agree, or strongly agree). Although the time taken to perform each task was also recorded, it was not used in weighing one environment’s superiority over another to avoid it being a confounding factor. The third element of the task analysis was a survey, one for each environment, which recorded the users’ overall experiences.

##### The Task Design for the Task Analysis

The participants were given three identical tasks to perform, common to each of the environments: the task of creating and viewing a data dictionary, editing an existing (the newly created dictionary in the first step) data dictionary, and standardizing the variable names of the data dictionary. Aiming for fair comparison, each task had an identical number of steps and method of performing the tasks, and the participants were instructed to follow these steps precisely. At the end of each task, participants were instructed to save the work and check for any loss of work during the save operation.

The first task of creating a data dictionary instructed them to start with an empty data dictionary and then add three variables and their constraint definitions. The second task of editing the data dictionary involved re-opening the data dictionary and adding and editing select variables. The third task (Textbox 1) was to use the variable name as a search key-phrase to search for a matching term from a controlled vocabulary. Each environment had a way of executing the search (block search for all variable names as well as searching for each variable name separately). Once the search result set was presented, each participant chose the best match and created a link between the variable name and a reference to the controlled vocabulary term. These steps made sure an informed valid term binding was created for each variable name.

The participants answered two questions at the end of each task to gauge if the environment allowed them to accomplish the task well and the satisfaction associated with accomplishing the task, according to their perceived understanding and expectation of the goal. Each question was measured using a 5-Point Likert scale [28] with increasing scores: strongly disagree (1, lowest score), disagree (2), neither agree or disagree (3), agree (4), and strongly agree (5, highest score). Question III was originally designed as part of the representation analysis, which was excluded from the scope of this usability study. It was designed to capture the level of difference between what each user expected from the system and what the user actually did to accomplish the goal. The results were computed with a focus on responses to Question II, rather than Question III.

##### Survey Design for the Task Analysis

In an effort to capture additional data that included a participant’s overall experience with an environment, we included a set of survey questions (Textbox 2) related to the organization of interface elements, robustness (failure and recovering from a failure, eg, error messages and navigation), auxiliary user interface elements, and easiness with which information about next step could be found.

Survey questions also included overall satisfaction and comfort in using the environments. Each of the 9 survey questions had a binary response: Yes or No. An absence of response was counted as a *No*. Each survey question was also assigned a weight to compute the weighted average score for each environment, in addition to an unweighted average score. At the end, each participant was asked to pick their favorite environment and to rank the environments as first, second, and third in the order of their preference.

The participants were encouraged to provide feedback and convey their experiences, which were recorded as comments. These comments were cataloged and provided useful insights and much sought-after features expected from environments to manage metadata definitions.

#### Statistical Methods

##### Function Analysis

The TURF framework metrics (domain function saturation: within-model and across-model) were calculated by using the proportions of functions to demonstrate the functional coverage of each environment. These metrics provide instant understanding of critical and overhead functions each environment implements.

##### Task Analysis

The Kruskal-Wallis test [27], which is a nonparametric method of testing, was chosen to perform the Analysis of Variance [29] for ranking the environments. This method was employed because we did not want to assume normality and our sample size was marginal for parametric testing. A significant Kruskal-Wallis test indicates that an environment is significantly different from others, but it does not indicate how it is different (better or worse). For our purposes, the mean score was determined to be adequate as a mark of overall user experience.

**Task details of Study Task 3: to standardize a variable using its name.**

**3. Searching & Binding Controlled Terminology Terms**

a) Open the data dictionary updated in previous task.

b) Search and Link all the cell values of ‘Name’ column only. This might involve searching for values that do not find matches.

c) Verify that all four values in of ‘Name’ column have been linked.

d) Save (if needed) and Close the data dictionary.

e) Please write (or tell the study conductor) answers to the following questions.

**Please record your feedback about this task in this particular environment:**

I. Task completion time: ________minutes _______seconds

II. The system allows me to accomplish the task well?

1. _____Strongly disagree
2. _____Disagree
3. _____Neither agree or disagree
4. _____Agree
5. _____Strongly agree

III. The system enables me to accomplish the task well, according to my perceived understanding and expectation of the goal?

1. _____Strongly disagree
2. _____Disagree
3. _____Neither agree or disagree
4. _____Agree
5. _____Strongly agree

**A set of survey questions with their assigned weights were given to the participants to assess their overall experience and level of satisfaction in each of the three environments.**

Q1. It was simple to use this system. (0.75)

Q2. I can complete my work quickly and efficiently with this system. (1.0)

Q3. It was easy to learn to use this system. (1.0)

Q4. The information provided with this system is clear. (1.0)

Q5. The organization of the information on the system screens is clear. (1.0)

Q6. Whenever I make mistake using the system, I recover easily and quickly. (1.25)

Q7. It is easy to find information I needed. (1.0)

Q8. I feel comfortable using this system. (1.5)

Q9. Overall, I am satisfied with this system. (1.0)

In the task analysis, there were 26 participants, as one of the participants withdrew before we could conduct the task analysis. As there were more than 2 groups (3 environments), it was useful to see contrasts among environments to precisely understand the performance of one environment compared with another.

To assess these pairwise system differences, we subsequently performed the chi-square test [30]. Pairwise chi-square tests were performed on the dichotomous outcomes in the same manner as we employed for the Likert ranked scores.

A scale transformation was performed prior to the statistical testing, on the Likert scale, from (1, 2, 3, 4, 5) to (−1, −2, 0, 1, 2), to use zero as the center response for better understanding of the results, as a response of three was neutral, below being negative and above being positive. In addition to statistical testing, we also describe our findings in a descriptive side-by-side display of responses for each of the questions.

## Results

### Overview

The results of this usability study not only confirm the usefulness of D2Refine over the other environments but also offer useful insights into potential feature requirements. The participants discussed their experiences with existing systems and workarounds that they had to take to overcome the lack of functionality and to get jobs done.

### Function Analysis Metrics

**Figure 5:**
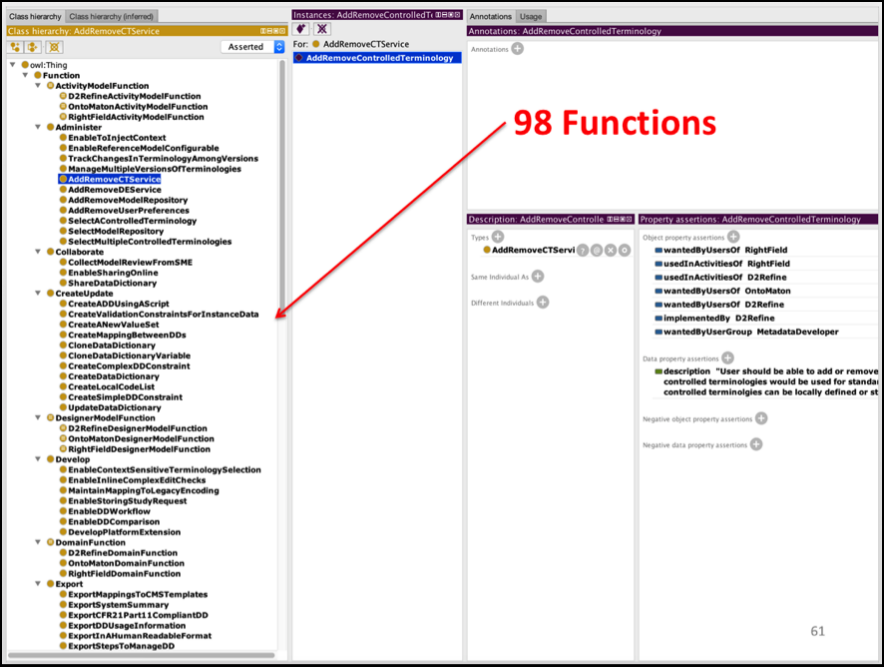
The Work Domain Ontology helped organize and catalog 98 distinctly identified functions.

After analyzing each participant’s responses to the user questionnaire and filtering the overhead functions, 98 distinct functions were identified. We used Protégé OWL Editor [31] to create the work domain ontology (Figure 5), where these functions were created as OWL classes and instances. The properties of OWL instances assisted storing membership of a function to its domain model and each participant’s responses.

**Figure 6:**
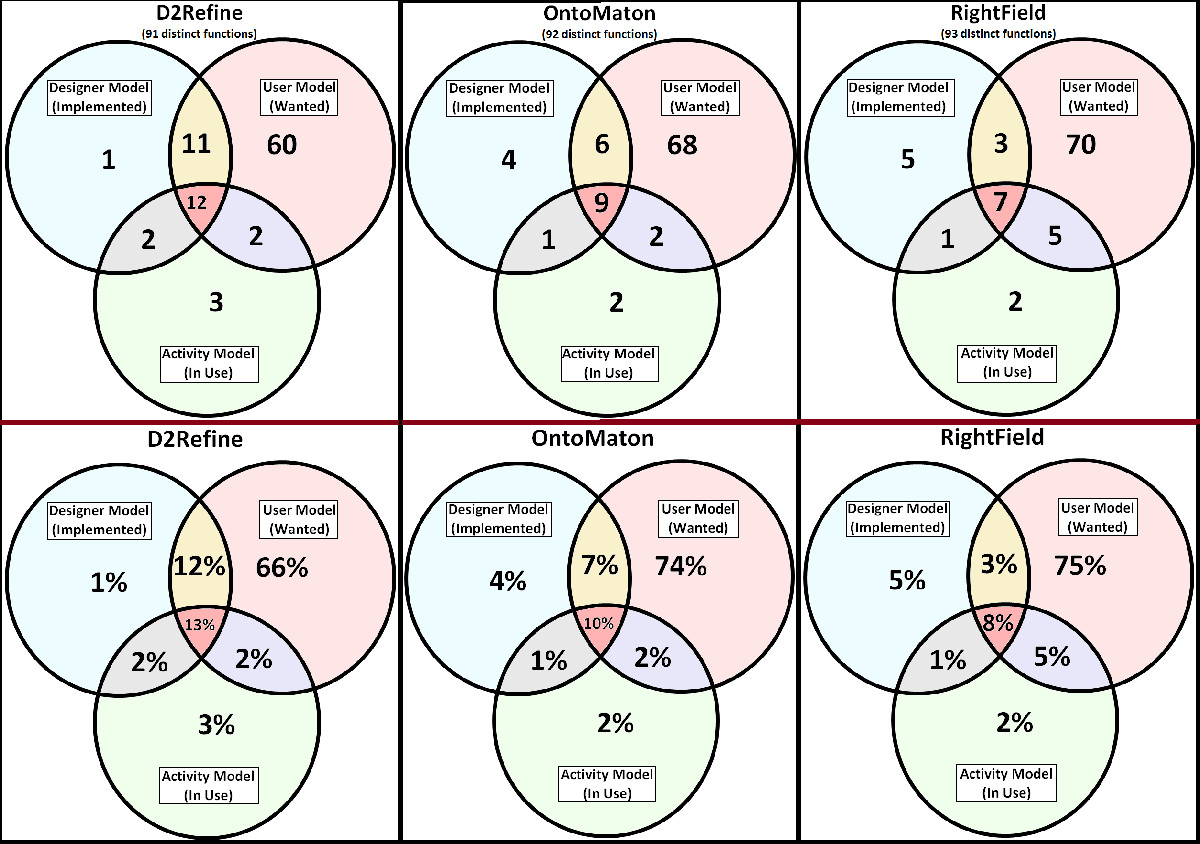
Venn diagrams of function coverage.

A Python-based utility queried this work domain ontology to calculate the TURF framework metrics and corresponding Venn diagrams to depict the function coverage. Figure 6 shows the function coverage for three environments. There were 91, 92, and 93 distinct functions for the three environments, respectively. There were 10 common functions that were implemented so far and task analysis employed these common functions like creating and editing data dictionaries. D2Refine had the largest number of functions in the overlapped areas of three models, indicating that testers favored its usefulness more, in comparison to OntoMaton and RightField. D2Refine also showed the least number of unused implemented functions. The results show that D2Refine had the most function saturation by implementing 25 out of 26 critical Designer Model functions compared with OntoMaton (implemented 16 out of 20 Designer Model functions) and RightField (implemented 11 out of 16 Designer Model functions). Both metrics of the TURF framework for the domain function saturation were calculated (Table 1) among the three platforms, where D2Refine had an edge over others.

The domain function saturation metrics also agreed with the observations of function coverage. It shows that overall, D2Refine implemented 28% of all functions, which was 7 percentage points better than OntoMaton and 11 percentage points than RightField. For the Within-Model domain function saturation, D2Refine had 96% saturation, which was 4 percentage points better than OntoMaton and 28 percentage points better than RightField.

Table 1 shows the TURF framework metrics of domain function saturation calculated according to their definitions in the sections “Function Analysis Metrics” and “The TURF Metrics” and Figure 4. D2Refine implemented 25 out of 26 Domain Model Functions, resulting in 96% coverage for Within-Model Function Saturation. The Across-Model Function Saturation was 28%, wherein D2Refine implemented 26 out of the 91 identified functions. Similar metrics were computed for OntoMaton and RightField.

**Table 1:** The TURF (task, user, representation, and function) framework metrics of domain function saturation calculated according to their definitions.

Domain function saturation	D2Refine		OntoMaton		RightField	
	N	n (%)	N	n (%)	N	n (%)
Within-model	26	25 (96)	20	16 (80)	16	11 (68)
Across-model	91	26 (28)	92	20 (21)	93	16 (17)

### Task Analysis Results

**Figure 7:**
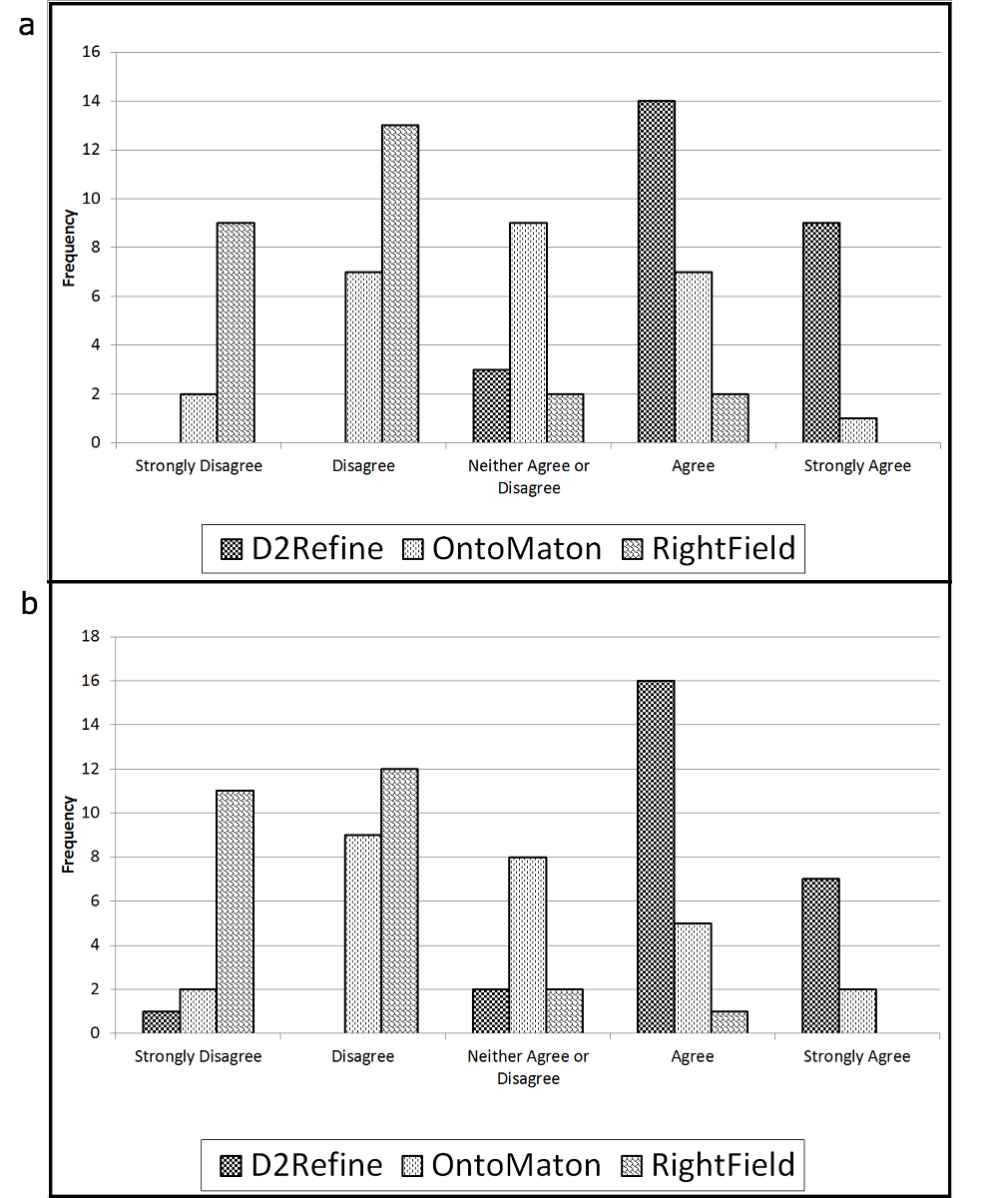
A side-by-side comparison of the three environments for Task 3 of searching a term and creating terminology binding for a variable name, showing a favorable trend for D2Refine: (a) The system allows me to accomplish the task well? and (b) The system enables me to accomplish the task well, according to my perceived understanding and expectation of the goal?

The results for the task analysis are shown in Tables 2 and 3. Table 2 shows when all three environments were compared for the three identical tasks and mean of their responses for the two questions were tallied. The statistics show that a statistically significant environment score was most helpful in accomplishing the given task well. Please note that while D2Refine was directly comparable with OntoMaton and RightField for Tasks 1 and 2 (creating and updating a data dictionary), it clearly stood out for Task 3 (*P*<.001, Kruskal-Wallis test). Figure 7 shows the participant’s responses to the Likert scale choices for the Task 3 questions, which were the most interesting part of the task analysis. Task 3 was nontrivial in all three environments, as it required searching for a matched term and creation of term binding. Both bar charts for Task 3 reflect the favorability of D2Refine over the other two environments.

Here, Table 2 shows the satisfaction level of participants for all three environments, compared during task analysis. The *P* values show significant differences and indicate a clear leaning toward D2Refine, especially for Task 3. Table 3 shows the pairwise comparison of D2Refine with OntoMaton and RightField. We observed significant differences for Task 1 and Task 2, indicating that the participants’ experiences were not significantly different for the tasks of creating and editing data dictionaries. However, the statistics showed strong significant differences for Task 3, the task of searching and linking cell values with controlled terminology terms. In other words, the *P* values in Table 3 indicate that D2Refine is significantly different from OntoMaton and RightField. Taking into account the statistical leaning toward D2Refine, as exhibited in Table 2, for Task 3, these significant differences indicate the favorability of D2Refine.

**Table 2:** Satisfaction level comparison of the three environments using the average score on Likert scale.

Task and questions		D2Refine	OntoMaton	RightField	*P* value^a^
**Task 1**					
	Question 1^b^	1.3	1.5	0.9	<.118
	Question 2^c^	0.8	1.4	0.7	<.014
**Task 2**					
	Question 1	1.5	1.5	0.8	<.036
	Question 2	1.2	1.4	0.6	<.017
**Task 3**					
	Question 1	1.2	−0.1	−1.1	<.001
	Question 2	1.1	−0.2	−1.3	<.001

^a^*P* values were calculated using Kruskal-Wallis test for a significance level of.05.

^b^Question 1: The system allows me to accomplish the task well?

^c^Question 2: The system enables me to accomplish the task well, according to my perceived understanding and expectation of the goal?

**Table 3:** Pairwise comparison between D2Refine and OntoMaton and RightField^a^.

Task and questions		Environment comparison	
		D2Refine vs OntoMaton	D2Refine vs RightField
**Task 1**			
	Question 1^b^	.05	.19
	Question 2^c^	.023	.56
**Task 2**			
	Question 1	.89	.027
	Question 2	.35	.041
**Task 3**			
	Question 1	<.001	<.001
	Question 2	<.001	<.001

^a^Statistics show significantly different experience for the tasks (especially Task 3); *P* values were calculated using Kruskal-Wallis test for a significance level of.05.

^b^Question 1: The system allows me to accomplish the task well?

^c^Question 2: The system enables me to accomplish the task well, according to my perceived understanding and expectation of the goal?

### Task Analysis Survey Results

The responses for the 9 survey questions were tallied for their mean scores. We also compared their overall choice for a favorite environment, if it were to be used by participants on regular basis. These results of the survey questions are listed in Table 4. Even though some statistics did not show significant differences (especially for Task 1 and Task 2) among the three environments, participants showed a strong leaning toward D2Refine for using it to create, update, and standardize the data dictionaries and other metadata creation needs. Here, we demonstrate significant differences with highly significant *P* values (*P*<.001) using the Kruskal-Wallis test for survey sum scores (weighted and nonweighted) and chi-square test for categorical choice for a favorite system.

**Table 4:** Side-by-side comparison of the three environments for the survey results (weighted and nonweighted) and for overall favorite environment.

Measure	D2Refine	OntoMaton	RightField	*P* value^a^
Nonweighted survey (mean score)	5.4	3.8	1.1	<.001^b^
Weighted survey (mean score)	5.8	4.1	1.1	<.001^b^
Participant’s favorite environment (participants)	17	9	0	<.001^c^

^a^*P* values were calculated using Kruskal-Wallis and chi-square tests for a significance level of .05.

^b^Kruskal-Wallis test.

^c^Chi-square test.

## Discussion

### Principal Findings

In the first prototype of D2Refine, we extended the OpenRefine platform to create and import data dictionaries and standardize them using a CTS2 Reconciliation Service. The aim was to determine D2Refine’s usability and effectiveness in managing data dictionaries. Our approach of conducting a moderated usability study in which D2Refine was compared with similar solutions was subsequently clarified. The TURF Framework of EHR Usability was a great tool for designing and planning this usability study. The participants were recruited from a group of interested individuals and included study developers, administrators, information technology professionals, or end users. All had adequate domain knowledge and familiarity with data dictionaries. The tutorials and introduction to these environments were carefully created to avoid favoring a particular environment. This helped us in reducing learning curve greatly for participants and minimizing any confounding factor like lack of domain knowledge.

The interface and workflow steps are almost identical in these three systems for obtaining a data dictionary, updating it, and standardizing its variables. While OntoMaton and RightField leverage the capabilities of Google Spreadsheets and Microsoft Excel, respectively, D2Refine leverages OpenRefine. Participants were given identical or equivalent empty spreadsheets to help carry out the steps. The structured and unstructured responses were gathered and used to calculate TURF metrics and perform statistical side-by-side comparisons.

This usability study provided much needed feedback and insight into the usefulness of D2Refine. The TURF Framework of EHR Usability proved to be a great tool to evaluate the usefulness of each of the participating environments. The function analysis questionnaire helped develop the work domain ontology and also identified 98 distinct functions for possible implementation. Function analysis metrics demonstrated significantly better function coverage (both within and across domain) for D2Refine, as compared to OntoMaton and RightField. Task analysis showed favorable significant differences for accomplishing the identified tasks with D2Refine, especially for term search create terminology binding. Participants’ feedback to survey questions and overall experiences favored D2Refine.

### Limitations

While the participants were able to complete their work in all three environments, there were some issues and errors participants faced and some issues translated into recommendations for future improvements. We have highlighted the participants’ observations, complaints, and wish-list items that were captured during the task analysis.

The process of typing in the variable definitions was relatively easier in OntoMaton and RightField because participants were directly working with the actual spreadsheets, whereas the D2Refine interface required additional clicks to navigate from one cell to another. Additional steps were needed to add blank rows for new variables in D2Refine. The participants noticed that none of the environments validated the values as they were typed in. The integrated metadata elements (to create and edit data dictionary) of D2Refine platform confused participants with the data dictionary metadata.

For some participants, OntoMaton failed to query and retrieve results, and for some searches, result set categorization was incorrect. Preserving and presenting the term binding details were confusing for OntoMaton and RightField environments. There was no guidance for users to make informed choices when creating term bindings, especially in the case of OntoMaton and RightField. In the case of D2Refine, users could see the term details from reusing the reconciliation service, but this D2Refine functionality could be improved.

The behaviors of interface elements of RightField were disappointing. The column width and font size were very small and cell values were lost due to nonstandard or incorrect interface implementation. There were numerous issues with loading multiple ontologies in RightField and working with them. RightField failed to load moderate to large size ontology, and partial load forced us to reset the working environment and resulted in lost work. RightField always lost the term binding details when data dictionaries reopened and hence heavily discouraged its use.

Although we selected common functionalities for comparison, there are other capabilities that each environment offered, in their own way. We did not include these additional capabilities in this study because they were not common across all three environments. However, two additional features of D2Refine (1) configurable CTS2 Reconciliation Service and (2) serialization of data dictionary into a standard format like openEHR ADL are worth mentioning here.

D2Refine has a built-in reconciliation service, configured to connect to NCI’s LexEVS CTS2 Service, which allows users to search for a term in controlled terminologies at NCI. Although the built-in connection service is similar to what OntoMaton and RightField offer, D2Refine lets users add any additional CTS2 compliant service end-point to its list of available reconciliation services. Although the capability of augmenting the reconciliation service for any CTS2-compliant representational state transfer server is not included in this usability study, D2Refine still proves its worth with its built-in reconciliation service, which is at least on-par with OntoMaton and RightField. D2Refine can also persist a data dictionary by serializing it into a standard format such as openEHR ADL [32], which enhances interoperability and makes it shareable and reusable.

Note that installing and configuring OpenRefine for D2Refine, like any other application, requires an additional step that users have to take before D2Refine can be used. This additional step might hinder D2Refine’s reach to a wider community, and hence, it forces us to replicate and integrate D2Refine into existing environments. At present, the environments of Microsoft Excel, iMedidata RAVE [33], and SAS Data Management Software [34] are the top choices of participants for starting and working with data dictionaries. These participants indicated their desire to extend these existing environments to avail the features of D2Refine. As a stand-alone application, D2Refine would still be greatly helpful as a complementary solution to ease the process of study design.

### Conclusions

The benefits of D2Refine’s simpler interface and reconciliation feature were validated by this usability study. Even though D2Refine is a prototype for performing data dictionary management, it compares favorably with other existing platforms and environments, which have been evolving over the recent years. The results of this usability study show clear interest and favorability toward the D2Refine platform. Participants not only wanted to see it develop but also to use it as an auxiliary solution that complements their work environment. This usability study provided valuable data as we evaluated our strategy for D2Refine and informed the improvement areas for future development. We believe that the outcome of this work will significantly improve the capabilities of existing informatics tools to manage heterogeneous clinical study data dictionaries and their standardization to improve semantic interoperability of the resulting data models. The artifacts including questionnaires, work domain ontology, and Python utility produced in this study are available online [35].

## Abbreviations

ADL: Archetype Definition Language
CTS2: Common Terminology Services
dbGaP: database of Genotypes and Phenotypes
EHR: electronic health record
ISA: Investigation, Study, and Assay
LexEVS: Lexical Enterprise Vocabulary System
NCI: National Cancer Institute
OWL: Web Ontology Language
TURF: task, user, representation, and function
